# Effects of Modified Graphene Oxide on Thermal and Crystallization Properties of PET

**DOI:** 10.3390/polym10060613

**Published:** 2018-06-04

**Authors:** Li Xing, Yao Wang, Shichao Wang, Yu Zhang, Sui Mao, Guanghui Wang, Jixian Liu, Linjun Huang, Hao Li, Laurence A. Belfiore, Jianguo Tang

**Affiliations:** 1 Institute of Hybrid Materials, The National Base of International Scientific and Technological Cooperation on Hybrid Materials, The National Base of Polymer Hybrid Materials in the Programme of Introducing Talents Dicipline to Universities, College of Materials Science and Engineering, Qingdao University, Qingdao 266071, China; xingliqdu@163.com (L.X.); wangsc@qdu.edu.cn (S.W.); zhagyulyn@163.com (Y.Z.); maosui001@163.com (S.M.); guanghuiwang@qdu.edu.cn (G.W.); ljx@qdu.edu.cn (J.L.); newboy66@126.com (L.H.); lihao0416178@163.com (H.L.); belfiore@engr.colostate.edu (L.A.B.); 2 Department of Chemical and Biological Engineering, Colorado State University, Fort Collins, CO 80523, USA

**Keywords:** PET, functionalized graphene oxide, thermal stability, crystallization property

## Abstract

In this article, graphene oxide nanosheets grafted with low molecular weight poly(ethylene terephthalate) were in situ synthesized via carboxylation, acyl chlorination and grafting modification in order to improve the compatibility between GO and PET phases and enhance the thermal stability and crystallization properties of PET. Fourier Transform Infrared (FTIR), X-ray Photoelectron Spectroscopy (XPS), and Atomic Force Microscopy (AFM) characterization results demonstrated that LMPET chains have been successfully grafted onto the surface of GO. To further investigate the influence of modified GO on properties of PET, modified PET was prepared by incorporating the GL-*g*-LMPET nanofillers into the PET matrix using the melt-blending method. Due to the similar polarity and strong interaction between LMPET and PET molecules, GL-*g*-LMPET nanofillers were homogeneously dispersed in PET matrix. Thermal properties and crystallization properties of obtained nanocomposites were systematically characterized using Differential Scanning Calorimetry (DSC), X-ray Diffraction (XRD), and Thermo Gravimetric Analysis (TGA). Results show that GL-*g*-LMPET nanofillers could improve the thermal stability of PET, e.g., increase up to 16.6 °C in temperature at the maximum rate of weight loss. In addition, the GL-*g*-LMPET also acts as an efficient nucleating agent for PET, exhibiting (1) higher crystallization temperatures; (2) higher degrees of crystallinity; and (3) faster rates of crystallization.

## 1. Introduction

As a kind of thermoplastic and semicrystalline polymer, Poly(ethylene terephthalate) (PET) is widely used in fields of fibers [1], packaging films [2] and engineering plastics [3] due to its chemical stability, resistance and barrier properties [4]. However, the insufficient thermal stability, slow crystallization and nucleation rate of PET limit its further application in specific fields [5]. For example, the benzene ring in the main chain of PET, while imparting rigidity, also causes slow crystallization during cooling, which will adversely affect the spinning process of high-speed fibers [6]. To solve the above-mentioned shortages, chemical modification and physical modification were applied to improve the performance of PET. Generally speaking, some drawbacks exist in organic modification, such as uncontrollable side reactions, harsh reaction conditions and complicated operation procedures. Compared with chemical modification, physical modification demonstrates relative priority due to its convenient operation and low cost. The addition of heterogeneous nanofillers nucleating agents can increase the crystallization rate of PET and obtain various properties at the same time [7,8]. Numerous nanoparticles, including clay, carbon nanotubes, TiO_2_, SiO_2_ and GO, have been incorporated into a PET matrix using the melt blending method [2,9,10]. 

Among all the nanoparticles, graphene oxide (GO) has attracted considerable research attention due to its excellent thermal stability [11], electrical properties [12], large specific surface area [13], room temperature quantum Hall effect [12], and superconductivity [14]. Many efforts have been made to incorporate GO into a PET matrix to prepare nanocomposites with high mechanical modulus [15,16] and outstanding electrical conductivity [17]. To date, only a few works have been reported to achieve good dispersion of GO in PET. Shim et al. prepared PET/unfunctionalized GO nanocomposites and found that GO nanosheets exhibited a nonuniform dispersion in PET matrix [18], which limits its further applications such as high pressure bottles. It is well known that the good dispersion of nanoparticles and the strong interfacial interaction with the polymer are two important factors to improve the physicochemical properties of nanocomposites [6]. However, due to the existence of a large number of nonpolar functional groups, GO presents poor compatibility with most organic solvents and water-insoluble polymers [16,19]. To solve the aggregation of GO sheets, chemical modification [20,21] of GO becomes an ideal method. After chemical modification, the physical and chemical properties of GO can be manipulated significantly. At the same time, the interfacial interaction and compatibility of GO in a polymer matrix can be also improved, which are conducive to preparing high-performance nanocomposites and achieving load transfer between GO and the polymer matrix [11,22]. Among all the chemical modifications of GO, in situ polymerization has been proved to be an effective way [23,24,25,26], which could avoid the agglomeration of GO during the modification process and enhance the interaction between GO and other components. Taking advantage of this, Yuan et al. found that the thermal stability of PP could be improved significantly by incorporating functionalized GO into the matrix [27]. Considering the increased dispersibility of functionalized GO in polymers as well as the improved properties of their nanocomposites, it is promising to achieve the promoted performance by incorporating surface-functionalized GO nanofillers into the PET matrix.

In this work, to improve dispersion and interfacial strength, functionalized GO nanosheets were synthesized via a three-step reaction through a new approach. GO was pre-modified with carboxylation and acylation and then grafted with low molecular weight poly(ethylene terephthalate) (LMPET) via in situ polymerization. Carboxylation reaction was used to provide more reactive sites and acylation reaction to obtain higher reactivity, thus achieving a complete surface modification of GO. The surface-functionalized GO nanosheets were further added into the PET matrix by melting blending to obtain the PET/GL-*g*-LMPET nanocomposites. Due to the fact that the polar parameter of LMPET grafted on the surface of GO was similar to that of PET matrix, it is beneficial to increase the interfacial compatibility between PET and GO phases, and eventually improve thermal stability and crystallization properties of PET. The structure of GL-*g*-LMPET as well as the thermal property and crystallization behavior of its composites were correspondingly discussed to provide comprehensive knowledge on the influence of modified GO on the PET matrix, providing fundamentals for the preparation of other high-performance composites using functionalized GO. 

## 2. Materials and Methods

### 2.1. Materials

Natural flake graphite was purchased from Qingdao Daewoo Graphite Co., Ltd. (Qingdao, China). PET was supplied by Sinopec Yizheng Chemical Fiber Research Institute, Yizheng, China, (MW = 3 × 10^4^ with a intrinsic viscosity of 0.676 dL/g). Other reagents used in this work were provided by Sinopharm chemical Reagent Co., Ltd. (Beijing, China) All of the regents were used as received without further purification.

### 2.2. Surface Modification of GO Nanosheets

The functionalization of GO was divided into three steps. Firstly, synthesis of carboxyl-functionalized graphene oxide (GH): 20.0 mg graphene oxide (>90% purity) prepared using Hummers method [28], 1.2 g sodium hydroxide and 1.0 g chloroacetic acid were added into aqueous solution successively. This reacted at 60 °C for 3 h under ultrasonic conditions. After that, the mixture was centrifuged and washed with deionized water until the solution reached a neutral level. The obtained product was dried at 60 °C under vacuum for 24 h. 

Secondly, synthesis of acyl chloride-functionalized graphene oxide (GL): 20.0 mg GH was added into benzene and treated under ultrasonic for 2 h. Then, 10 mL thionyl chloride was added under the protection of nitrogen flow. The mixture was heated to 65 °C and reacted for 6 h. Finally, the resultant solution was washed using benzene to remove the non-reactive thionyl chloride in a high-speed centrifuge. Based on the original feed ratio of GO nanofiller and thionyl chloride matrix, the resulting GL mass fraction was determined to be 9.4 wt %.

**Figure 1:**
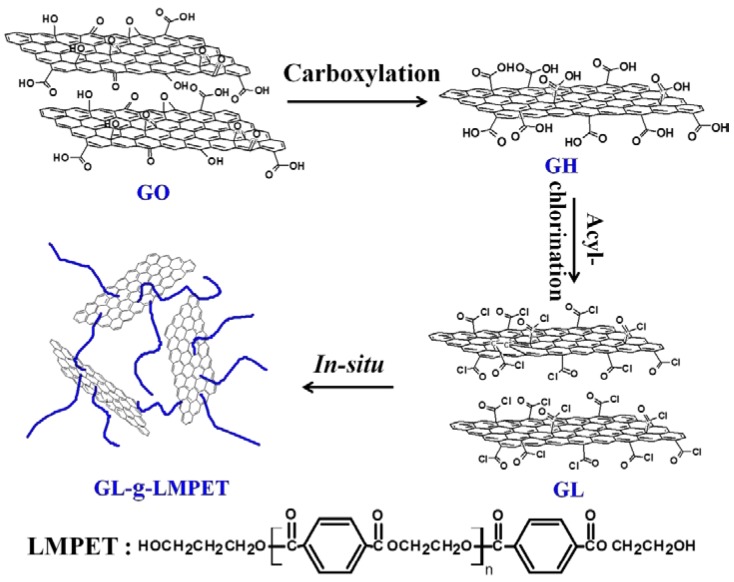
Schematic representation GL-*g*-LMPET nanoparticles formation.

Lastly, preparation of GL-*g*-LMPET: 11.2 g DMT (dimethyl terephthalate), 7 mL EG (ethylene glycol) and a small amount of zinc acetate were mixed in a three-necked flask and reacted under 190 °C for 2 h. After that, trace amounts of Sb_2_O_3_ used as catalyst and 0.5–1 drops of triphenyl phosphate acted as thermal stabilizer were added into the mixture, and further stirred for 1 h followed by the addition of 10 mg GL. The mixture was then heated to 230 °C and allowed to remain for 2 h. The obtained mixture was centrifuged and washed using phenol/carbon tetrachloride (*m:m* = 1:1) to remove ungrafted LMPET. Finally, the product was dried under vacuum at 80 °C for 24 h. Additionally, graphene oxide—LMPET (GO-*g*-LMPET) was prepared using a similar method (Figure 1) using GO without functionalization.

### 2.3. Melt-Blending of PET and GL-g-LMPET 

PET/GL-*g*-LMPET nanocomposites were prepared by a melt blending method using a HAAKE Rheometer (Shanghai, Chain) under 285 °C. The in situ modified graphene oxide nanosheets and PET were fully melt-blended, and the content of GL-*g*-LMPET nanosheets in the matrix was 0.5 wt %. For comparison, pure PET, PET/GO and PET/GO-*g*-LMPET nanocomposites were prepared according to the above procedure described for PET/GL-*g*-LMPET nanocomposites.

### 2.4. Characterization

Fourier transform infrared (FTIR) spectra were recorded on a Nicolet 5700 FTIR spectrometer (Varian, Inc., Palo Alto, CA, USA) over the range of 4000–500 cm^−1^. X-ray photoelectron spectra (XPS) were obtained from an ESCALAB250Xi-XL electron spectrometer (London, UK) at a using 150 W Al Kα radiation. A surface microstructure of GO nanoparticles was observed in a high resolution transmission electronic microscope (HR-TEM, JEM-2100CXLL, Akishima, Japan). Atomic force microscopy (AFM, Agilent 5400, Akishima, Japan) was used to determine the thickness of GO and modified GO at ambient temperature. Surface morphology analysis of PET and its nanocomposites were carried out by means of a scanning electron microscope (FE-SEM, JSM-7500F microscope, Akishima, Japan). Contact angle measurement (CAM) and surface free energy estimation of the nanocomposites were carried out at room temperature on a JY-82 contact angle system (New Castle, DE, USA). The average contact angle from six different locations on each nanocomposite was determined and the experimental uncertainty was within ±2°. X-ray diffractometer (XRD, Rigaku, Kyoto, Japan, λ = 0.154 nm) was used to test the crystalline properties of PET/GL-*g*-LMPET nanocomposites. The scanning was ranged from 5° to 60° with scanning speed of 2 (°)/min. DSC (821e Mettler-Toledo, Zurich, Switzerland) was used to characterize the thermal properties of PET based nanocomposites. Initially, samples were heated to 300 °C with a ramp rate of 10 °C/min and kept for 5 min to remove the thermal history. Then, samples were cooled and re-heated from 25 to 300 °C with a ramp rate of 5/10/15/20 °C/min. The crystallization and melting data were obtained from second cooling and heating curves. Thermogravimetric analysis (TGA) was performed on a PerkinElmer Diamond thermal analyzer (Waltham, MA, USA). Samples were heated from 50 to 600 °C nitrogen with a ramp rate of 10 °C /min under a continuous nitrogen flow rate of 20 mL/min. 

The degree of crystallinity (*X_C_*) for the pure PET and nanocomposites were determined from the DSC traces by the enthalpy variation during the second melting scan using the following Formula (1):

*X_C_* = Δ*H_m_*/(1 − *m*)Δ*H*_0_, (1)

where Δ*H_m_* is the enthalpy of melting of the measured sample, *m* is the quantity of GL-*g*-LMPET, and Δ*H*_0_ is the enthalpy of melting of 100% crystalline PET, reported to be 140 J/g [29]. 

The relative crystallinity (*X_t_*) of PET and its composites at different times calculated according to Formula (2):

(2) $$X_{t}=\int_{t_{0}}^{t}(\frac{dH_{C}}{dt})dt/\int_{t_{0}}^{\infty }(\frac{dH_{C}}{dt})dt,$$

where *dH****_C_****/dt* is the heat flow rate, *t*_0_ and *t*_∞_ are the time at which crystallization starts and ends, respectively.

## 3. Results and Discussion

### 3.1. Structural Characterization of GL-g-LMPET

**Figure 2:**
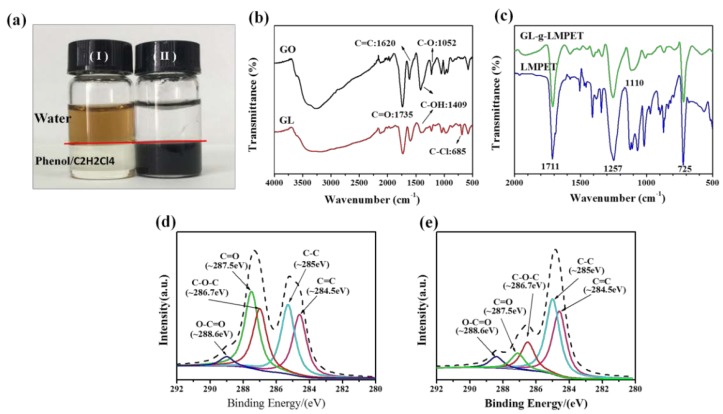
(**a**) solubility of GO (I) and GL-*g*-LMPET (II) in H_2_O (up)/ Phenol and C_2_H_2_Cl_4_ (down) solution; (**b**) FTIR spectra of GO and GL; (**c**) FTIR spectra of GL-*g*-LMPET and LMPET; XPS spectra (C1s) of GO (**d**) and GL-*g*-LMPET (**e**).

The dispersion of GO and GL-*g*-LMPET (0.5 mg/mL) in water and Phenol/C_2_H_2_Cl_4_ solution after 3 min of the sonication process were presented in Figure 2a. Due to the poor miscibility between H_2_O and Phenol/C_2_H_2_Cl_4_, a clear interface was observed (as marked with a red line). Since a large number of hydrophilic groups exist on the surface of GO, GO showed a homogeneous dispersity in aqueous phase (as shown in Figure 2a(I)). After grafting modification, the surface of GO was covered with LMPET chains, which could be well dispersed in its ideal solvent (Phenol/C_2_H_2_Cl_4_) [30]. Therefore, the dispersion of GO transferred from H_2_O to Phenol/C_2_H_2_Cl_4_ after chemical modification. This phenomenon could be an indicator to some extent of the successful grafting of LMPET chains onto the surface of GO nanosheets [31]. 

To further confirm the structure of modified GO, FTIR was applied to characterize the structure of GO and modified GO, as shown in Figure 2b,c, respectively. In the case of GL, the intensity of 3407 cm^−1^ (O–H stretching vibration), 1409 cm^−1^ (O–H deformation vibration) and 1052 cm^−1^ (epoxide groups stretching vibration) [11,27] decreased a lot after chemical modification, indicating that the content of OH groups and epoxide groups decreased after the carboxylation reaction. Compared with the FTIR spectra of GO and GL, significantl signals attributed to –C–Cl (O=C–Cl) appeared at 685 cm^−1^, suggesting the successful reaction between COOH groups and acyl chloride. Based on the data, GL-*g*-LMPET was further synthesized and the FTIR spectrum was shown in Figure 2c. Two peaks at 1257 and 1711 cm^−1^ corresponding to C–O–C and C=O vibrations of PET [32,33] appeared after the grafting modification, demonstrating that LMPET chains have been grafted on the surface of GO. Moreover, the appearance of significantly enhanced peaks at 1110 and 725 cm^−1^ indicates the C–H bond vibration of the benzene ring [33]. The above results confirm the successful covalent grafting modification between O=C–Cl functional groups of GL and hydroxyl groups of LMPET. All samples involved in the above discussion were centrifuged and dissolved repeatedly to ensure the complete removal of influence from physical adsorption. To further investigate the structure of GL-*g*-LMPET, XPS was used to measure the near-surface composition and examine the valence states of the observed elements. C1s spectra of GO and GL-*g*-LMPET are shown in Figure 2d and Figure 2e, respectively. Five types of carbon can be found in GO, namely C=C (284.5 eV), C–C (285.0 eV), C–O–C (286.7 eV), C=O (287.5 eV) and O–C=O (288.6 eV) [16,23]. After functionalization, the intensity of C-O-C and C=O peaks (Figure 2e) decreased significantly, while that of the O–C=O peak increased a lot, suggesting that LMPET molecules have been grafted onto the surface of GO via covalent bond during in situ polymerization. 

**Figure 3:**
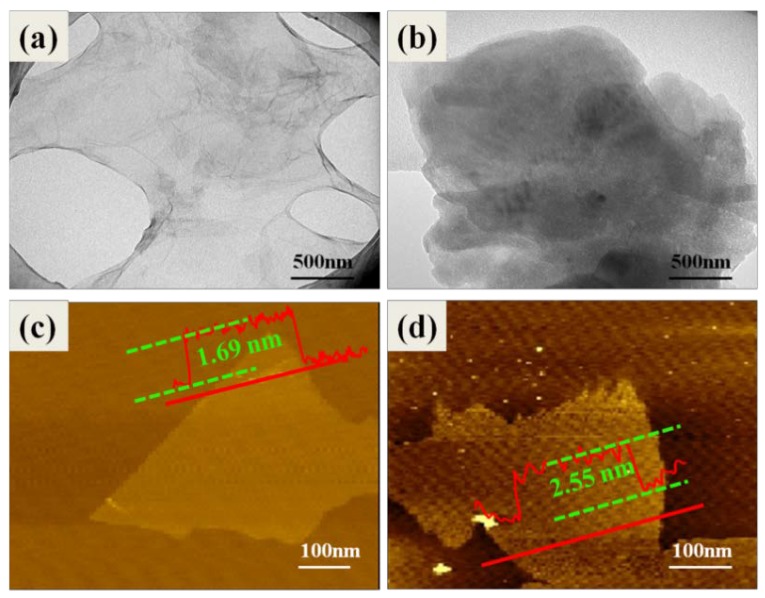
TEM images of (**a**) GO and (**b**) GL-*g*-LMPET; AFM image of (**c**) GO and (**d**) GL-*g*-LMPET.

TEM was utilized to investigate the microstructure of GO and GL-*g*-LMPET. Figure 3a reveals a transparent, clean surface of GO with some thin ripples. As is reported in previous literature, folds are an inherent property of graphene sheets, owing to the instability of the two-dimensional plane [34]. After functionalization, the TEM image of GL-*g*-LMPET is entirely different as shown in Figure 3b. We can clearly observe that the surface of GO sheets is covered by a thin coating, and this morphology is similar to the case of polymer-functionalized graphene or carbon nanotubes [35,36], which can be attributed to the LMPET chains grafted onto GO sheets, forming polymer grafting interface structures. Moreover, it is worth noting that the slightly aggregated structures of GL-*g*-LMPET sheets can be also observed in Figure 3b, which can be attributed to two main reasons: (1) strong π–π stacking between layers have a non-negligible attraction to each other, and (2) each LMPET chain has two terminal hydroxyl groups that would induce LMPET chains to inevitably graft onto two GOs. In short, the polymer interface layer can be successfully created. To further characterize the morphology of GO before and after modification, AFM was applied to characterize the thickness of GO and GL-*g*-LMPET monolayers [11]. As seen in Figure 3c,d, the thicknesses of GO and GL-*g*-LMPET were 1.09 and 1.65 nm, respectively. Thus, it has also been confirmed that the functionalization process has changed the morphology of GO sheets.

**Figure 4:**
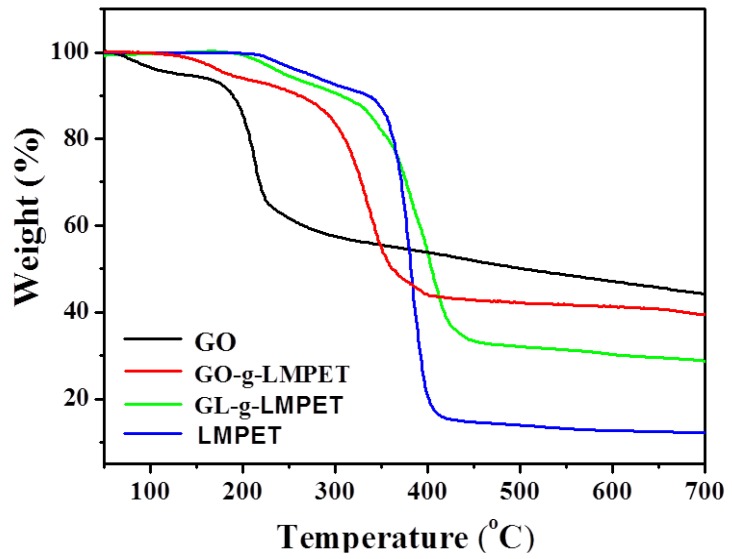
TGA profiles of GO, GO-*g*-LMPET, GL-*g*-LMPET, LMPET under N_2_ atmosphere.

The grafting content of LMPET was characterized using TGA, as shown in Figure 4. The weight loss of GL-*g*-LMPET can reflect the grafted LMPET molecular chains content to a certain degree due to the fact that the synthesized nanosheets were Soxhlet extracted for 72 h to remove unreacted polymers. For the TGA curve of GO, the initial mass loss appeared at around 100 °C, mainly attributing to the evaporation of water molecules adsorbed on the surface of GO. The major weight loss of GO appeared at 150~200 °C, which was caused by the pyrolysis of GO sheets [37]. However, it is observed that the major weight loss of the GO-*g*-LMPET and GL-*g*-LMPET are completed at 270~370 °C and 350~430 °C, respectively, which occurs later than that of the GO, illustrating that the grafting LMPET is effective for enhancing the thermal stability of GO [38]. Moreover, based on the char residue of GO (45 wt %), GO-*g*-LMPET (40 wt %) and GL-*g*-LMPET (27 wt %) under 700 °C, the weight percent of grafted LMPET onto GL and GO nanosheets were roughly calculated as 18 and 5 wt %. The comparatively higher grafting content of GL-*g*-LMPET may be benefited from the high active precursors (GL with multiple reactive sites).

### 3.2. Dispersion of Various Nano-GO into the Polymer Matrix

**Figure 5:**
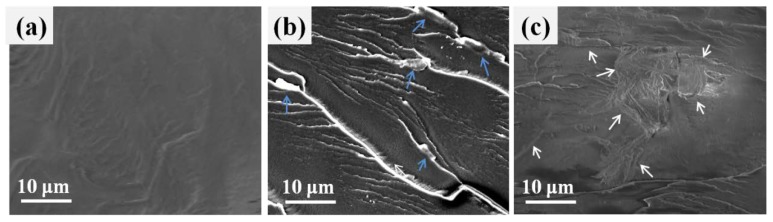
SEM images of PET (**a**), PET/GO (**b**) and PET/GL-*g*-LMPET (**c**) fractured surface.

It is well known that the good dispersion of nanoparticles and the strong interfacial interaction are two important factors to improve the physicochemical properties of nanocomposites modified polymer. SEM was applied to investigate the dispersion and interface of various nano-GO into the polymer matrix. Figure 5 shows the freeze-fractured surfaces of PET nanocomposites obtained at liquid nitrogen temperature. The roughness of all nanocomposites fractured surface is relatively higher compared with that of pure PET. Aggregates can be clearly seen in Figure 5b, indicating inhomogeneous dispersion of GO in PET matrix. In addition, some obvious gaps are observed on the surface (Figure 5b, blue arrows). This phenomenon can be explained that the interfacial interaction between the GO and the PET matrix is not ideal. By comparison, the PET/GL-*g*-LMPET nanocomposites in Figure 5c show that many smaller dimples on the fracture surface and GL-*g*-LMPET sheets can be well dispersed in the matrix, as no large agglomerates can be found. This demonstrates that the interface between GL-*g*-LMPET sheets and the PET matrix is stronger than that in the PET/GO and PET/GO-*g*-LMPET nanocomposites.

**Figure 6:**
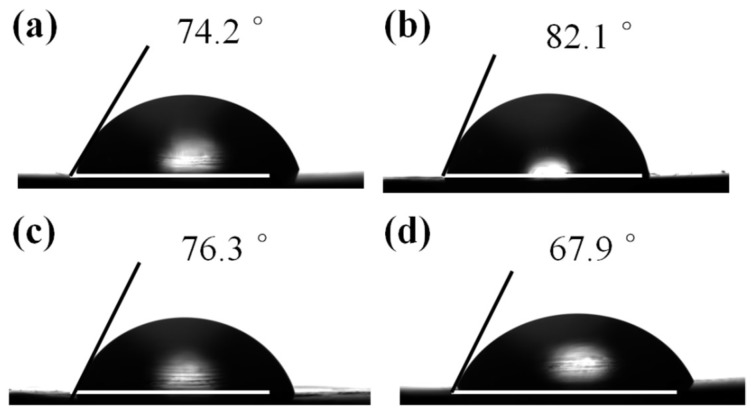
Pictures of water droplets on various nanocomposites: (**a**) pure PET; (**b**) PET/GO; (**c**) PET/GO-*g*-LMPET; and (**d**) PET/GL-*g*-LMPET.

We further used contact angle measurement to characterize the dispersion of nanoparticles on the surface of nanocomposites. Herein, we applied water probe solutions to study the dispersed (γ^D^) surface free energies of various nanocomposites, which are presented in Table 1. Figure 6 shows photos of water droplets on pure PET, PET/GO, PET/GO-*g*-LMPET and PET/GL-*g*-LMPET nanocomposites. In contrast to PET/GO and PET/GO-*g*-LMPET nanocomposites, the GL-*g*-LMPET particles embedded in the PET matrix significantly decreased the average contact angle of the probe liquids. This shows that the functionalized GO nanoparticles can increase γ^D^ of the PET nanocomposite. In other words, unfunctionalized GO and imperfect functionalized GO-*g*-LMPET have a strong tendency to agglomerate because of high surface free energy, resulting in less level dispersion in the PET matrix. However, the surface-functionalized GO nanoparticles process similar polarity with PET, which is beneficial to increase the interfacial compatibility and alter the orientation of the PET chains at the surface of nanocomposite, thus resulting in an increase in the γ^D^ of the resulting nanocomposites. In short, surface-functionalized GO nanoparticles tend to reside in the polymer body [39,40].

### 3.3. Influence of Various Nano-GO on Thermal Stability and Crystallization Properties of PET

**Figure 7:**
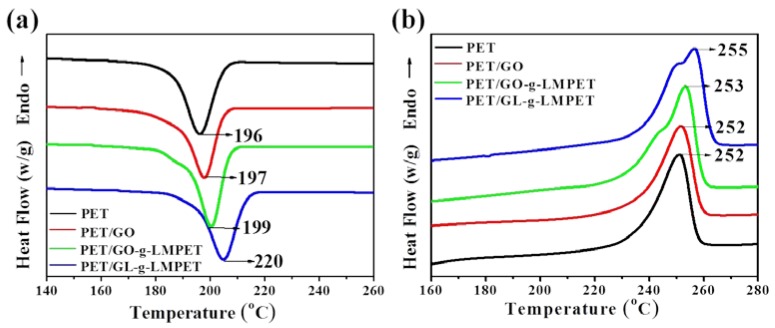
The heating curve (**a**) and the cooling curve (**b**) of pure PET, PET/GO, PET/GO-*g*-LMPET and PET/GL-*g*-LMPET at the rate of 10 °C·min^−1^.

The effect of different nucleating agents on thermal behavior of PET matrix during heating and cooling processes was characterized by DSC [41,42], as shown in Figure 7. The corresponding data were listed in Table 2. From Figure 7a, we can find that the melting temperature (*T*_m_) of PET/GL-*g*-LMPET nanocomposites notably increased as compared with those of pure PET, PET/GO and PET/GO-*g*-LMPET, whereas the PET/GO and PET/GO-*g*-LMPET only performed trivial changes. Figure 6b depicts the heating process of PET nanocomposites. Due to the nucleation effect of various nano-GO, the perfection degree of PET crystals increased, leading to the melting temperature increase. Interestingly, double melting peaks appeared when GL-*g*-LMPET was added into the PET matrix. The lower melting temperatures of PET nanocomposites were similar with that of PET, suggesting that the lower *T*_m_ was caused by the crystals of PET. For the higher melting temperature (*T*_m_), the perfection degree of crystals was relatively high, which may be caused by the crystals of PET/ GL-*g*-LMPET. Moreover, SEM of GL-*g*-LMPET can further clarify the two different kinds of PET crystals in nanocomposites (Figure 5d). GL-*g*-LMPETs were dispersed homogeneously in the PET matrix (as pointed at by white arrows), demonstrating a strong interaction between GO nanosheets and PET matrix. Thus, two different regions appeared in the PET matrix, namely GO surrounded by PET molecular and PET without GO. The former region formed crystals with higher perfection due to the strong interaction between GO and PET moleculars compared with that of the PET region, leading to the appearance of two different melting temperatures. 

**Figure 8:**
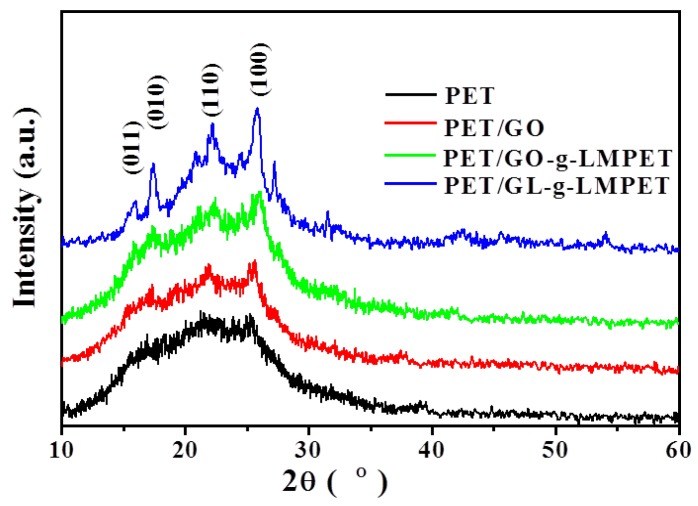
XRD curves of pure PET, PET/GO, PET/GO-*g*-LMPET and PET/GL-*g*-LMPET.

The XRD patterns provide important insight into the effects of GO, GO-*g*-LMPET and GL-*g*-LMPET on the crystalline structure of PE in Figure 8. It is known that nanofillers have a significant effect on the crystallization ability of semi-crystalline polymers [43,44]. Thus, various nano-GO, as the heterogeneous nucleus, was introduced in the present work. Compared to pure PET, the four sharp peaks appeared at 2θ ≈ 16.4°, 17.8°, 22.8° and 26.1° on the XRD curves of PET/GO, PET/GO-*g*-LMPET and PET/GL-*g*-LMPET, corresponding to the (011), (010), (110) and (100) diffraction planes, respectively [30]. This is clear proof for improved crystallinity of the nanocomposites. In addition, the intensity of diffraction peaks is gradually enhanced with the nanofillers addition of GO, GO-*g*-LMPET and GL-*g*-LMPET. It indicates that GL-*g*-LMPET acts as a kind of more high-effect nucleating agent to improve the crystallization ability of PET. To further investigate the crystallinity of PET and PET nanocomposites, the relative crystallinity *X*_C_ was calculated by Equation (2) and listed in Table 2. The results obtained agree with the outcome of XRD, the PET/GL-*g*-LMPET nanocomposite exhibits the optimal crystallization behavior and its crystalline increased by 6% more than pure PET. 

**Figure 9:**
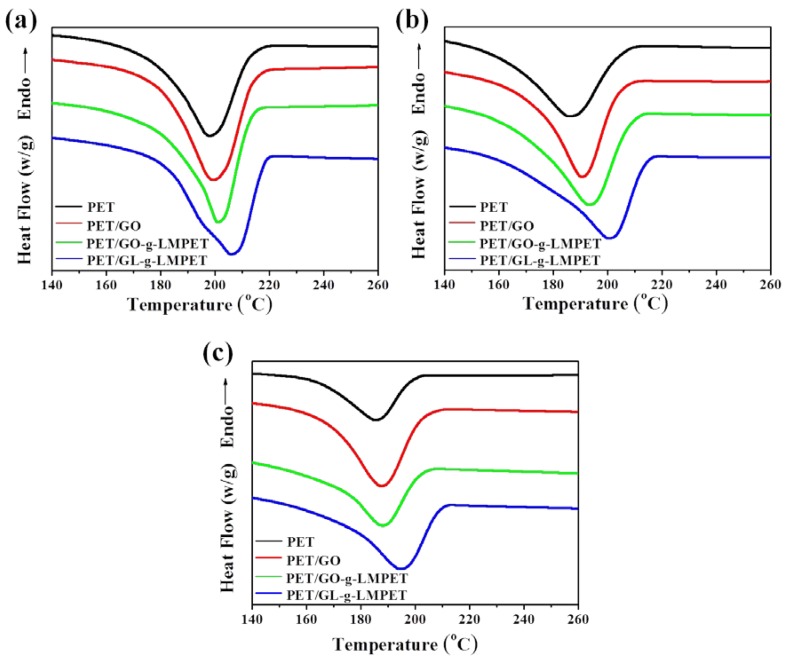
DSC curves for pure PET, PET/GO, PET/GO-*g*-LMPET and PET/GL-*g*-LMPET at different cooling rates. (**a**) 5 °C·min^−1^; (**b**) 15 °C·min^−1^ and (**c**) 20 °C·min^−1^.

**Figure 10:**
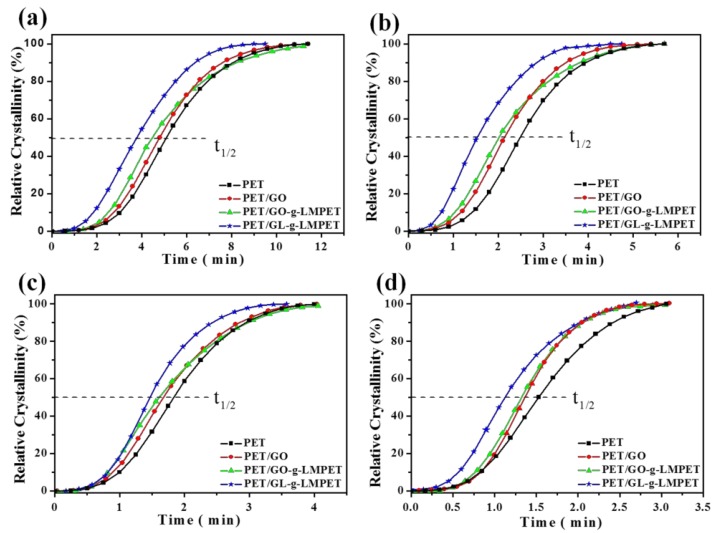
Plots of relative degree of crystallinity (*X*_t_) versus crystallization time (*t*) for pure PET, PET/GO, PET/GO-*g*-LMPET, and PET/GL-*g*-LMPET at different cooling rates. (**a**) 5 °C·min^−1^; (**b**) 10 °C·min^−1^; (**c**) 15 °C·min^−1^ and (**d**) 20 °C·min^−1^.

Another criterion commonly used to evaluate crystallization property is the crystallization rate of nanocomposites. DSC of all the samples at different cooling rates were performed and relevant results are presented in Figure 7a and Figure 9. At the same cooling rate, Tmc shifted to higher values gradually with the addition of the nucleating agents GO, GO-*g*-LMPET and GL-*g*-LMPET. Based on the above DSC crystallization curves and Formula (2), non-isothermal crystallization process of PET and its composites at different cooling rates were obtained and shown in Figure 10. Moreover, we adopted an important parameter, the value of t_1/2_, to characterize the crystallization rate (the time at which the relative crystallinity of the polymers achieves 50% of the total crystallinity measured at that temperature and can reflect the overall crystallization rate of the polymers), and the lower t_1/2_ values indicate higher crystallization rate. Obviously, at the same cooling rate, the t_1/2_ values decreased in the order of PET/GO, PET/GO-g-LMPET and PET/GL-*g*-LMPET. PET/GL-*g*-LMPET achieving the higher crystallization rate is attributed to the good dispersion and strong interfacial adhesion of GL-*g*-LMPET in PET.

**Figure 11:**
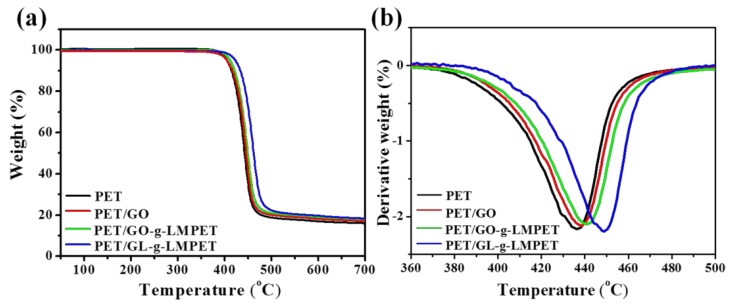
(**a**) TGA curves of pure PET, PET/GO, PET/GO-*g*-LMPET, and PET/GL-g-LMPET; (**b**) DTG curves of pure PET, PET/GO, PET/GO-*g*-LMPET, and PET/GL-*g*-LMPET.

The degradation behavior of pure PET and its nanocomposites were investigated by TGA and relevant results, which are presented in Figure 11. Obviously, compared to pure PET, the initial degradation temperature of all PET/GL-*g*-LMPET samples were at higher temperatures (Figure 11a) compared with that of PET, and PET/GL-*g*-LMPET nanocomposites exhibit the highest degradation temperature. Another criterion commonly used to evaluate thermal stability is the maximum thermal decomposition temperature (*T*_max_). The *T*_max_ of PET/GO, PET/GO-*g*-LMPET and PET/GL-*g*-LMPET nanocomposites were increased by approximately 5.3, 7.4 and 16.6 °C, respectively (Figure 11b). The improvement of thermal stability of PET/GL-*g*-LMPET is much more obvious than other nanocomposites, which benefits from the physical barrier effect of GL-*g*-LMPET and delays the escape of degradation products [45]. Additionally, possessing similar polarity, LMPET and PET chains interacted with each other, improving the compatibility between the GO and PET matrix, forming comprehensive interfacial entanglements, and prohibited polymeric chains from slipping over each other in the melting process [46,47]. 

## 4. Conclusions

In this research, a novel strategy to significantly enhance thermal stability and crystallization properties of PET was demonstrated. Carboxylation and acyl chlorination provide more reactive points and promote the grafting reaction of LMPET, achieving a complete surface modification of GO. In addition, the similar polarity of LMPET and PET contribute to improving the dispersion and interface interaction of GO in a polymeric matrix. Due to the addition of GL-*g*-LMPET, which acted not only as the efficient nucleating agent for PET crystallization, but also as a physical barrier during the thermal degradation of the polymer, the thermal stability and crystallization property of nanocomposites were significantly enhanced. Specifically, a 16.6 °C enhancement of the temperature was observed at maximum thermal decomposition temperature (*T*_max_), and the crystallization temperature was also increased by 9 °C along with the crystallinity (*X*_C_) increment up to 29.7%. Moreover, the crystallization rate (*X*_t_) of PET/GL-*g*-LMPET nanocomposites was simultaneously enhanced. This work represents a novel and effective functionalization strategy to improve the dispersion and interface interaction of GO in a polymeric matrix and brings significantly enhanced properties, therefore facilitating industrial applications of PET in a wider range.

## Figures and Tables

**Table 1:** Contact Angle and surface free energies of Water on various nanocomposites.

Sample	Average contact angle (°)	γ^D^
Pure PET	74.2	39.2
PET/GO	82.1	34.1
PET/GO-*g*-LMPET	76.3	37.7
PET/GL-*g*-LMPET	67.9	43.2

**Table 2:** Thermo-performance parameters and the degree of crystallinity of various nanocomposites.

Sample (0.5 wt %)	*T*_mc_ (°C)	*T*_m_ (°C)	Δ*T* (°C)	Δ*H*_m_ (J/g)	*X*_C_ (%)
PET	196	252	56	33.2	23.7
PET/GO	197	252	55	34.7	24.9
PET/GO-*g*-LMPET	199	253	54	35.9	25.8
PET/GL-*g*-LMPET	205	255	50	44.2	29.7
